# Proceedings of the Buffalo Medical Association

**Published:** 1879-06

**Authors:** Joseph N. Fowler

**Affiliations:** Secretary


*ART. II.—Abstract of the Proceedings of the Buffalo Medical Association, April 2*, 1879.—*Post-partum Hemorrhage.* By Prof. J. P. WHITE. Reported by JOSEPH N. FOWLER, M. D., Secretary.

The President, Dr. THOMAS LOTHROP, occupied the chair. The minutes of the last meeting were read and adopted.

Prof. JAMES P. WHITE spoke upon the subject of Post-partum Hemorrhage, as follows:

*Mr. President and Gentlemen:*

When you were kind enough to invite me to address you on the subject of Post-Partum Hemorrhage, it was in view of its great importance, and not because I hoped to throw any new light upon the subject that I consented.

GOUCH says that before passing the threshold of the Parturient chamber the medical practitioner should be prepared to encounter uterine hemorrhage in all its forms. The danger of hemorrhage after the delivery of the child is very great, and the means of foreknowing it absolutely *nil.* There is no time to consult books or arrange for a consultation. You perceive, therefore, how important it is that we should be prepared at all times to encounter it. A brief delay may find our patient beyond assistance. The amount of blood lost must be estimated by the impression made upon the system, rather than the quantity. The pulse is the earliest and best test of danger. If the hemorrhage be severe the pulse becomes increased in frequency and diminished in force. Dimness of vision, ringing in the ears, vomiting, coldness of the extremities, a clammy perspiration, are all valuable indications of exhaustive hemorrhage. Convulsions ordinarily supervene before syncope and death take place. The exhaustion brought on from hemorrhage differs from that due to the process of labor. The pulse not only flags, but there is always a death-like palor.

What are some of the causes of this accident? The chief and most common is uterine inertia, atony or non-contraction of the uterus. Laceration of the uterine neck or vagina, or of the womb itself, are among the causes.

You are all familiar with the appearance of the uterine placenta surface. So soon as the placenta becomes detached if the uterus remains uncontracted the valveless vessels are permitted to pour out their contents into the uterus, and a most alarming hemorrhage ensues, and it is only by the contractile power of the womb that they are closed, and ligated, as it were. And upon this alone must we depend for arrest of the flow. Diathesis has much to do as a cause. Feeble persons are more liable to hemorrhage than others. Exhausting diseases, in which the coagulability of the blood is lessened; tumors of all kinds; fibroids within the cavity of the uterus—or intra mural which increase its vascularity and prevent contraction; inversion of the uterus, whether partial or complete. The former I believe not infrequently present without our knowledge of it. Hemorrhage may occur either before or after delivery of the placenta. A protracted labor, if followed by the rapid expulsion of the child at its termination. Over distention of the uterus, which may lessen its contractility, increasing the size of the vessels as well. A hot climate predisposes to hemorrhage. English ladies who go to India are liable to this accident. The hemmorrhage may be concealed, the blood accumulating in the uterine cavity, gradually dilating and increasing it.

In treating post-partum hemorrhage we know better now how to manage it successfully than formerly. It may nearly always be prevented by faithfully following prophylactic measures. Always secure contraction of the uterus as the head of the child passes out, by placing the hand over the hypogastrium and manipulating the womb. Ergot should be administered at this stage. Forceps deliveries are more apt to be followed by hemorrhage, because of the rapidity of the child's expulsion, or withdrawal at the conclusion of the second stage. Rapid completion of this stage, or sudden evacuation of the uterus are frequent causes. Under such circumstances the advancement of the child should be retarded at the vulva, that the womb may have time to regain its tonicity and contract firmly. If there be any indications of relaxation of the organ at the conclusion of the second stage the ergot should be repeated. I hardly know in what form to recommend its administration. Squibb's and Wyeth's fluid extracts are good, but I prefer that the practitioner should gather his own ergot, run it through a coffee mill and prepare it himself at the time. After the flowing has commenced the pillows should be removed and the foot of the bed elevated so as to keep the patient's head low. Grasp the uterus with one hand and make firm pressure. Should not be in too much of a hurry to apply the bandage, it would only be in the way. Wait till the uterus has contracted under the hand and remains so for a considerable time before applying it. It should consist of some elastic material, such as flannel.

It is important to see that the bladder is evacuated. At this stage it is necessary to keep the patient quiet as well as depress her head, as there is great danger from exertion. Freely ventilate the room and maintain an equable temperature. Manipulation of the uterus is of the utmost importance. Seize it with the hand and hold it firmly. Failing in these measures apply cold to the hypo-gastrium. If it be convenient gather some snow in a napkin and apply it. Perhaps you may conclude to introduce ice into the vagina. If these measures do not succeed expose the abdomen and dash cold water on it. Avoid too protracted application of cold. A rubber blanket may be placed under the patient and cold water poured from a coffee-pot or pitcher. The object aimed at is shock. While this is going on continue manipulation and pressure. Ergot must again be resorted to. If the stomach rejects it, or you desire a more rapid effect, give a hypodermic injection of the solid extract dissolved in a little water. If you do not succeed with these agents administer opium and brandy freely. Opium does not paralyse the uterine fibre, but stimulates it to contract, and acts as a hæmostatic. If the cold douche is not followed by contraction of the womb alternate with hot water. The cold douche rarely fails. Our whole reliance is upon uterine contraction.

After the uterus has contracted and the placenta peeled off, the latter should be removed at once. The same rule holds true in concealed hæmorrhage. If the after-birth be retained pass the hand into the uterus, gently detach it, knuckle the uterine walls to excite contraction and remove it, while with the other hand external pressure should be made. If the whole of the placenta cannot be secured, remove that part which can be readily detached, but never resort to violence. One of the most powerful means of inducing uterine contraction is the ether spray directed upon the hypogastric region. Avoid too long continued use of cold as it will depress the vital energies. These are the remedies usually resorted to.

In extraordinary conditions, where there is irregular or hour-glass contraction follow up the cord, dilate the contracted fibres,detach the placenta, and let both hand and placenta be pushed out by the uterus as it contracts. The same rule applies to the removal of coagula.

In former times, and, indeed, in my own time, the tampon was used and the mouth of the uterus plugged up. It is plainly percieved that blocking up the mouth of the uterus with a tampon would dilate that organ and enlarge the orifices of the bleeding vessels and increase the danger. I have some confidence in applying the child to the breast as a means of exciting uterine contraction. We all know the sympathy existing between the two. This can be done, but must not interfere with other measures.

Styptic injections have been recommended, such as ferri persul —of which Barnes has been a special advocate for the last ten years. My own experience is rather opposed to its use. It coagulates the blood, and in this condition may enter the blood and produce death. Waving this objection it forms an incinderated mass, as it were, objectionable in many ways.

In Havana where this accident is said to be common, tincture of iodine is used in the same manner.

Dr. Mack, of St. Catherines, throws brandy into the uterus, and believes it superior to all other known remedies.

At the last meeting of the Gynecological Society Dr. Penrose advocated the application of vinegar to the interior of the uterus with a sponge, and thinks that when properly employed no patient should be lost. I have used vinegar as a styptic for many years, and keep it conveniently at hand to stop bleeding when examining and operating, in preference to all other styptics.

Dr. Playfair, in the last *Obstetrical Journal of Great Britain*, speaks highly of hot water injected into the uterus. All of these measures act by stimulating the womb to contract. I am reluctant, under any circumstances, to allow myself to inject the uterus.

Another method of treatment consists in making pressure upon the abdominal aorta, by pressure over the abdomen or by introducing the hand into the vaginal cul-de-sac, or uterus itself, and still more recently by passing the hand into the rectum. The last procedure I would be a little disinclined to adopt.

Galvanism may stimulate the womb to contract better than any other means, but unfortunately, a battery is rarely at hand on such occasions. The hypodermic injection of ether in dram doses when a patient is in a moribund state is a powerful stimulant.

Transfusion is useful under such circumstances.

Eschmark's elastic bandage applied to the lower extremities to diminish the amount of blood circulating in them and relieve cerebral exhaustion has also been employed.

I have spoken upon some of the most important points of a subject attended with greater danger and demanding more energetic means of relief than any other accident I ever met in my professional life. I have confined my remarks to hemorrhage after the delivery of the child, and omit any consideration of another interesting feature, the after-treatment.

Prof. J. F. MINER said he had no reply to make to so earnest and eloquent discussion of the subject involved. I have never had much fear or anxiety about hemorrhage succeeding labor. During my practice of thirty-two years, I have attended a large number of lying-in-women, and do not recollect losing a single patient from post-partum hemorrhage. The doctor impresses us with the great danger attending these cases, and I am gratified that I should have been so fortunate. I never have resorted to uterine injections, or electricity—have used cold douche, and often introduced the hand into the cavity of the womb, and if coagulum was found drawn it out. I question the possibility of grasping the dilated uterus with the hand and forcing it to contract permanently, and am not prepared to accept the position taken by authors, that external manipulation is so important. The womb is made to contract by shock, and I doubt the efficacy of any measures other than those calculated to stimulate this function of contraction.

Dr. CRONYN said he had scarcely any thing to add. The object of Dr. White's address was to point out the very great danger of post-partum hemorrhage and the measures to prevent and control it. The Professor omitted to mention one form of hemorrhage which follows labor, notwithstanding perfect contraction of the uterus has been secured. In these cases it is evidently due to laceration of the cervix, which might be arrested by the application of vinegar and other styptics as suggested by the Doctor. It is here that the tampon, vinegar and hot water are useful. During many years' experience in practice it has been my misfortune to meet several cases of post-partum hemorrhage of a very alarming character, which caused me much anxiety. I highly value the method of grasping the uterus with the hand and holding it forcibly. I recollect one woman whom I attended in three labors, all of which were followed by alarming hemorrhage. On the last occasion her husband was able by the power of his grasp to keep the womb contracted and saved her life. It was necessary to continue this for three hours. These cases are frightful and sometimes prove fatal.

After the transaction of some miscellaneous business, and the election of the following officers:

*President*—LUCIEN HOWE.

*Vice-President*—W. W. MINER.

*Secretary*—JOSEPH FOWLER.

*Treasurer*—F. E. L. BRECHT.

*Librarian*—J. B. SAMO.

On motion, the Association adjourned.

